# The BDSF quorum sensing receptor RpfR regulates Bep exopolysaccharide synthesis in *Burkholderia cenocepacia* via interaction with the transcriptional regulator BerB

**DOI:** 10.1038/s41522-022-00356-2

**Published:** 2022-11-22

**Authors:** Elisabeth Steiner, Rebecca E. Shilling, Anja M. Richter, Nadine Schmid, Mustafa Fazli, Volkhard Kaever, Urs Jenal, Tim Tolker-Nielsen, Leo Eberl

**Affiliations:** 1 Department of Microbiology, University of Zürich, Zürich, Switzerland; 2 Costerton Biofilm Centre, Department of Immunology and Microbiology, Faculty of Health Sciences, University of Copenhagen, Copenhagen, Denmark; 3 Research Core Unit Metabolomics, Hannover Medical School, Hannover, Germany; 4 Focal Area of Infection Biology, Biozentrum, University of Basel, Basel, Switzerland; 5 Present Address: Department of Infectious Diseases, Robert Koch Institute, Berlin, Germany

**Keywords:** Biofilms, Pathogens, Bacteriology

## Abstract

The polysaccharide Bep is essential for in vitro biofilm formation of the opportunistic pathogen *Burkholderia cenocepacia*. We found that the *Burkholderia* diffusible signaling factor (BDSF) quorum sensing receptor RpfR is a negative regulator of the *bep* gene cluster in *B. cenocepacia*. An *rpfR* mutant formed wrinkled colonies, whereas additional mutations in the *bep* genes or known *bep* regulators like *berA* and *berB* restored the wild-type smooth colony morphology. We found that there is a good correlation between intracellular c-di-GMP levels and *bep* expression when the c-di-GMP level is increased or decreased through ectopic expression of a diguanylate cyclase or a c-di-GMP phosphodiesterase, respectively. However, when the intracellular c-di-GMP level is changed by site directed mutagenesis of the EAL or GGDEF domain of RpfR there is no correlation between intracellular c-di-GMP levels and *bep* expression. Except for *rpfR,* deletion mutants of all 25 c-di-GMP phosphodiesterase and diguanylate cyclase genes encoded by *B. cenocepacia* showed no change to *berA* and *bep* gene expression. Moreover, bacterial two-hybrid assays provided evidence that RpfR and BerB physically interact and give specificity to the regulation of the *bep* genes. We suggest a model where RpfR binds BerB at low c-di-GMP levels to sequester this RpoN-dependent activator to an RpfR/RpfF complex. If the c-di-GMP levels rise, possibly by the enzymatic action of RpfR, BerB binds c-di-GMP and is released from the RpfR/RpfF complex and associates with RpoN to activate transcription of *berA*, and the BerA protein subsequently activates transcription of the *bep* genes.

## Introduction

The second messenger bis-(3′–5′)-cyclic dimeric guanosine monophosphate (c-di-GMP) is commonly used by bacteria to control the transition between motile and sessile lifestyles, with high c-di-GMP levels promoting the biofilm mode of growth. Intracellular levels of c-di-GMP are controlled through the opposing activities of diguanylate cyclases (DGCs), which catalyze the synthesis of c-di-GMP from two GTP molecules, and phosphodiesterases (PDEs), which catalyze cyclic di-GMP degradation to pGpG. DGCs are characterized by the presence of GGDEF domains, and PDEs are characterized by the presence of either EAL or HD-GYP domains (reviewed in Jenal 2017^1^). Enzymes involved in c-di-GMP turnover are present in large numbers in many bacterial species, raising the intriguing question of how signaling specificity is achieved. Temporal sequestration of c-di-GMP signaling would require the tightly regulated expression of c-di-GMP metabolic enzymes or its controlled turnover. Functional and spatial sequestration, on the other hand, could be achieved through multiprotein complexes that are maintained through specific protein–protein or protein–DNA interactions, thus creating ‘microcompartments’ as discussed in Hengge^2^.

*Burkholderia cenocepacia* H111^3^ is a member of the *Burkholderia cepacia* complex (BCC), a group of more than 25 related bacterial species that can cause life-threatening disease in immunocompromised and cystic fibrosis (CF) patients^4–6^. In this strain, biofilm formation and the expression of virulence factors are controlled by two quorum sensing (QS) systems: (i) the CepIR system, which depends on an *N*-acyl homoserine lactone (AHL) signal molecule, and (ii) the RpfFR system, which relies on *cis*-2-dodecenoic acid, referred to as *Burkholderia* diffusible signal factor (BDSF)^7^. While CepIR represents a classical QS system where the CepR-AHL complex binds to specific DNA sequences in the promoter regions of target genes^8^, little is known about the signal transduction pathway downstream of the RpfFR system. This system consists of the BDSF synthase RpfF^9^, an enoyl-CoA hydratase, and the BDSF receptor RpfR^10^, a multidomain protein containing a PAS, a GGDEF, and an EAL domain. Recent work has shown that RpfR and RpfF form a heterohexameric protein complex consisting of three RpfF and three RpfR monomers^11^. Upon binding of BDSF to RpfR, the PDE activity of RpfR is stimulated and the cellular c-di-GMP level is reduced. Inactivation of either *rpfF* or *rpfR* resulted in a reduction in swarming motility, proteolytic activity, virulence, and biofilm formation in microtiter trays^10^. Mapping of the BDSF stimulon by RNA-Seq and shotgun proteomics identified various genes or proteins, including the large surface protein BapA and the lectin operon *bclACB*, that are responsible for several of the observed mutant phenotypes, and showed that the BDSF- and AHL-dependent regulons partially overlap^12^. Interestingly, when the intracellular c-di-GMP concentration in *B. cenocepacia* H111 is artificially elevated, expression of QS-regulated genes are suppressed and, consequently, the virulence of the strain is attenuated^13^.

Previous work has shown that c-di-GMP controls the production of the exopolysaccharide (EPS) Bep in *B. cenocepacia* H111 by stimulating the activity of the transcriptional regulator BerA^14,15^. Expression of BerA is dependent on the alternative sigma factor RpoN and the bacterial enhancer binding protein BerB, which is activated by c-di-GMP^16^. Bep was recently shown to be a water-insoluble polysaccharide that consists of the tetrasaccharide repeating unit [3)-α-d-Gal*p*-(1 → 3)-α-d-Glc*p*-(1 → 3)-α-d-Gal*p*-(1 → 3)-α-d-Man*p*-(1 → ]_*n*_^17^. Bep is essential for wrinkled macrocolony morphology on nutrient agar plates, pellicle formation in standing cultures, and biofilm formation in flow-through chambers^15^.

In this study, we show that RpfR is a negative regulator of the Bep biosynthesis cluster and provide evidence that RpfR is not only able to degrade but also to synthesize c-di-GMP in vitro as well as in vivo. For full functionality of RpfR both the GGDEF and the EAL domain of the protein have to be intact. Our data suggest that the specificity of RpfR-controlled Bep expression is dependent on a specific interaction of RpfR with BerB rather than changes in the global cellular c-di-GMP pool.

## Results

### Inactivation of the RpfFR QS system causes a wrinkled macrocolony morphology by inducing expression of the Bep EPS gene cluster

**Fig. 1:**
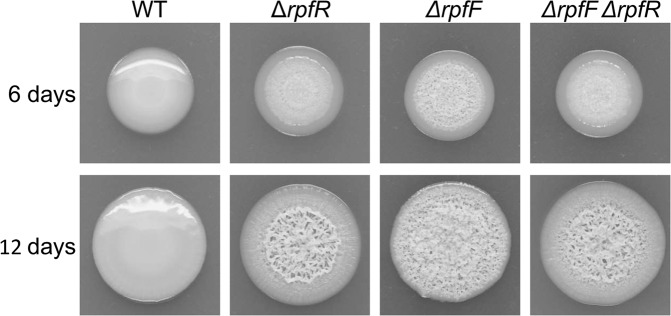
Colony morphology of *B. cenocepacia* strains with mutated or intact RpfFR QS system. Cell suspensions of wild-type *B. cenocepacia* H111 (WT) and mutants carrying an in-frame deletion of *rpfF*, *rpfR*, or both genes were spotted on NYG agar plates. Colony morphology was assayed after growth for 6 and 12 days.

We observed that *rpfR* and *rpfF* in-frame deletion mutants formed wrinkled colonies on NYG agar plates, whereas wild-type *B. cenocepacia* H111 colonies were smooth (Fig. 1), suggesting that RpfR is a negative regulator of a compound that causes the wrinkled colony morphology. Macrocolony wrinkling of *rpfF* or *rpfR* mutants was virtually indistinguishable from the wild-type strain on AB minimal medium supplemented with glucose or glycerol (Supplementary Fig. 1). For this reason, we used NYG medium for determining macrocolony morphology throughout this study. In an attempt to identify additional genes involved in the regulation of the wrinkled colony phenotype, we screened a transposon mutant library generated in the wild-type background for colonies displaying the wrinkled colony phenotype on NYG agar plates. Out of the approximately 40,000 mutants screened, 19 displayed wrinkled colony morphology. Sequencing revealed that all 19 mutants carried transposon insertions at different positions within the *rpfR* gene, emphasizing the involvement of RpfR as a negative regulator of a structural component causing the wrinkled colony morphology. It is worth noting that *rpfF* mutants are unlikely identified in this experimental set-up, as the *rpfF* defect can be rescued by surrounding colonies releasing BDSF.

Previous work has identified GtrR (BCAL1536) as a downstream regulator of the RpfFR system^18^. Inactivation of this gene was shown to affect motility, biofilm formation, and virulence. However, the colony morphology of a *gtrR* in-frame deletion mutant was found to be indistinguishable from that of the wild-type strain, suggesting that this regulator is not involved in the regulation of the wrinkled colony phenotype.

**Fig. 2:**
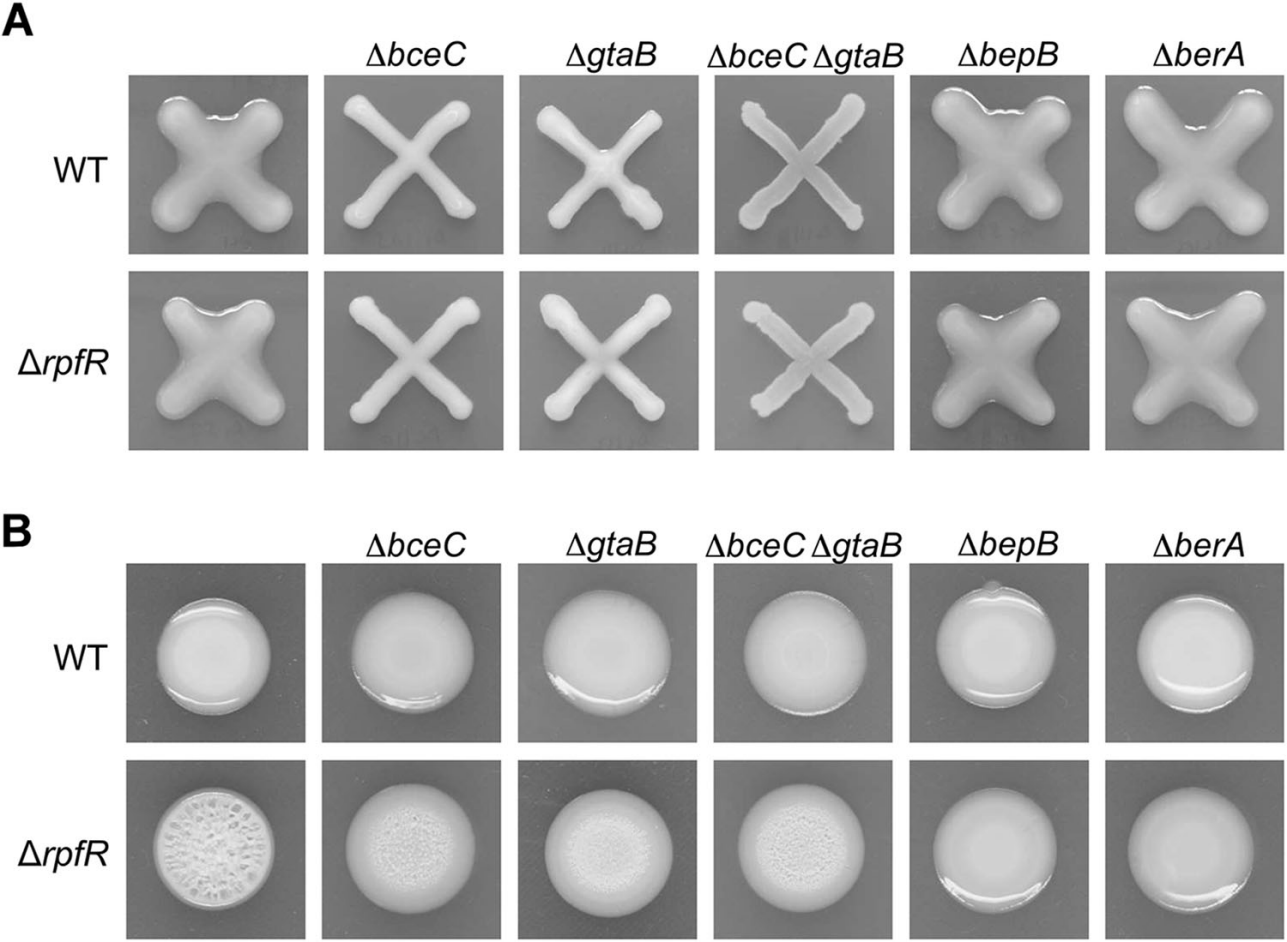
Assessment of cepacian production and role of cepacian and Bep in colony morphology. **A** Assessment of cepacian production of wild-type and mutant strains after growth on YEM agar plates for 2 days. Cepacian production appears as slime surrounding the cross-colonies of the bacteria that are not mutated in the *bceC* or *gtaB* genes. **B** Role of cepacian and Bep in colony morphology of wild-type and *rpfR* mutants. Candidate EPS gene clusters were inactivated in the wild-type (upper panels) and the *rpfR* mutant background (lower panels). The Bep cluster was inactivated by deleting *bepB* (Δ*bepB*) or the BerA regulator (Δ*berA*). Cepacian production was reduced/abolished by deleting *bceC* (Δ*bceC*), *gtaB* (Δ*gtaB*) or both genes (Δ*bceC* Δ*gtaB*). Colony morphologies after growth on NYG agar plates for 6 days are shown.

EPS often affects colony morphology and we, therefore, anticipated that the wrinkled colony morphology of the *rpfR* mutant could be caused by overexpression of EPS, which is often controlled by the second messenger c-di-GMP^10,16^. The *B. cenocepacia* H111 genome harbors gene clusters involved in the biosynthesis of at least three EPS molecules: cepacian, cellulose, and the recently identified *Burkholderia* exopolysaccharide, Bep. We first investigated if cepacian is involved in the *rpfR* mutant wrinkled colony morphology. Previous work has shown that the biosynthesis of cepacian is directed by the two gene clusters *bce-I* and *bce-II*^19^ and is induced by sugar alcohols such as mannitol in many *Burkholderia* strains^20^. To disable cepacian biosynthesis, in-frame deletion mutants of *bceC* and/or *gtaB* were generated. The *bceC* gene is part of the *bce-I* cluster and *gtaB* is part the of the *bce-II* cluster. In-frame deletions of these genes were generated in the wild-type strain as well as in the Δ*rpfR* mutant. To confirm that cepacian production had indeed been abolished in the constructed strains, the wild-type as well as single, double and triple mutants were streaked on YEM plates to assay for mannitol-induced cepacian production. As shown in Fig. 2A, cepacian production was significantly reduced in the single mutants Δ*bceC* and Δ*gtaB* and completely abolished in the Δ*bceC* Δ*gtaB* double mutant, indicating that both cepacian clusters *bce-I* and *bce-II* are required for high-level cepacian production. Inactivation of *rpfR* had no apparent effect on cepacian production, suggesting that RpfR is not involved in the regulation of this EPS molecule, in agreement with a recent transcriptome analysis^21^. Phenotypic characterization of the strains on NYG agar plates revealed that inactivation of cepacian production (Δ*rpfR* Δ*bceC*, Δ*rpfR* Δ*gtaB*, and Δ*rpfR* Δ*bceC* Δ*gtaB)* did not abolish the wrinkled phenotype but led to flatter and smoother macrocolonies relative to the Δ*rpfR* mutant, suggesting that cepacian contributes to the architecture of microcolonies but is not the actual cause of wrinkle formation.

We then investigated if the *rpfR* wrinkled colony morphology is caused by overexpression of Bep. Production of Bep was disrupted by deleting *bepB*, a gene that is essential for Bep EPS biosynthesis^15^, or *berA*, which encodes a c-di-GMP responsive CRP/FNR family transcriptional regulator that controls expression of the *bep* gene cluster^14,15^. Inactivation of either *bepB* or *berA* reverted the wrinkled phenotype of the Δ*rpfR* mutant back to the smooth and shiny wild-type morphology (Fig. 2). This suggests that the Bep EPS is responsible for the wrinkled morphology of Δ*rpfR* colonies and that RpfR is a negative regulator of Bep production.

### RpfR represses expression of the *bepA-N* gene cluster

**Fig. 3:**
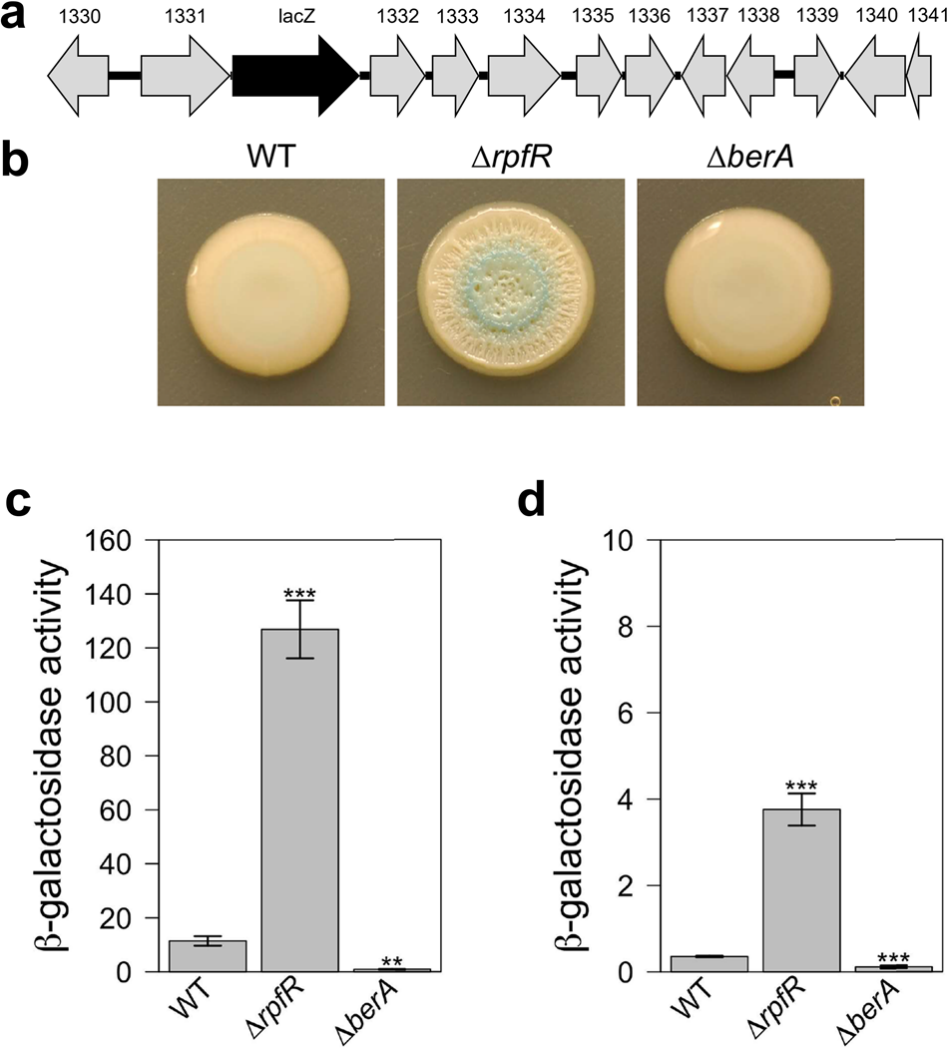
Effect of RpfR on Bep expression. A *lacZ* reporter gene was introduced into the chromosome downstream of the BCAM1331 (*bepB*) gene by allelic exchange in the wild type, the *rpfR* mutant and the *berA* mutant. **A** Schematic presentation of the location of the *lacZ* reporter gene into the *bep* operon between *bcam1331* and *bcam1332*. **B** Macrocolony morphology and β-galactosidase expression of the *bepB*::*lacZ* reporter strains after growth for 6 days on NYG agar plates supplemented with X-gal. **C, D** Quantification of β-galactosidase activity (Miller units) of *bepB*::*lacZ* reporter strains grown in AB medium supplemented with 1.5% glycerol (**C**) or NYG medium (**D**) for 24 h. Note that the scale is different in panels (**B**) and (**C**). The values shown are the means ± SEM of 2 independent experiments. The significance levels, as determined using a one-way ANOVA, of the difference between the strains are indicated above the bars; ***P < 0.001; **P < 0.01.

To investigate the role of RpfR in the expression of the *bepA-N* cluster, we inserted a promoterless *lacZ* gene downstream of *bepB* (*bcam1331*) in the wild-type and Δ*rpfR* background such that expression of the other genes in the operon was unaffected (Fig. 3A). To quantify *bepB* expression, reporter strains were grown in liquid AB medium supplemented with 1.5% glycerol as carbon source (ABGly) (Fig. 3C) or in NYG medium (Fig. 3D). Under both culture conditions, *bepB* expression was about 10-fold increased in the Δ*rpfR* mutant relative to the wild-type background. It is worth noting, however, that the overall level of β-galactosidase activity was about 30 times higher in ABGly than in the NYG medium (Fig. 3C, D). Given that the wrinkled macrocolony phenotype was mainly observed in the NYG medium (Fig. 1, Supplementary Fig. 1), these results suggest that the high level of *bep* expression differs between NYG and AB minimal media and that cultures grown in liquid cultures or on solid media are not directly comparable. To visualize *bep* expression in macrocolonies, we inoculated the strains on NYG agar plates supplemented with X-gal. After 6 days of incubation, β-galactosidase activity was observed in macrocolonies of the Δ*rpfR* mutant but not of the wild-type. The fact that the Δ*rpfR bepB::lacZ* strain still displayed a wrinkled colony morphology confirmed that the insertion of *lacZ* did not interfere with the expression of genes downstream in the operon (Fig. 3A). Interestingly, *bep* expression was not uniform within the macrocolony but was found to be constrained to a circular inner region of the colony (Fig. 3B), indicating that only a subpopulation of the colony produced Bep EPS. When expression of Bep was boosted by providing berA in trans on a plasmid, we not only observed extensive wrinkling but also that β-galactosidase activity was concentrated within the ridges of the wrinkled colony (Supplementary Fig. 2), suggesting that localized expression of Bep EPS is the actual cause of macrocolony wrinkling.

**Fig. 4:**
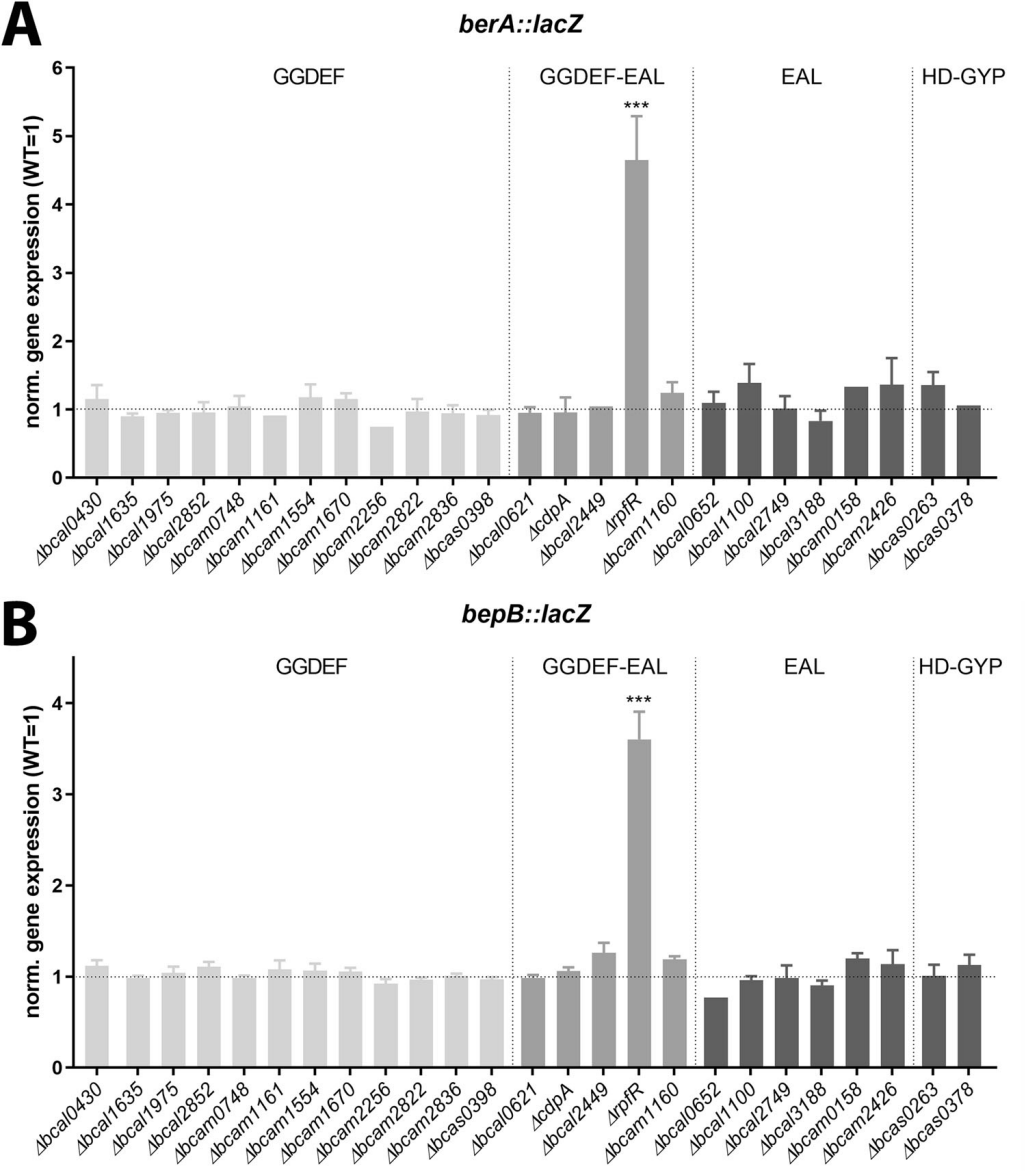
Expression of *berA*::*lacZ* (A) and *bepB*::*lacZ* (B) reporter genes in *B. cenocepacia* DGC/PDE mutant cultures. Light gray bars represent mutants of genes encoding GGDEF-only proteins, gray bars represent mutants of GGDEF-EAL proteins, whereas dark gray bars represent mutants of EAL-only and HD-GYP proteins. β-galactosidase activities are normalized to the level of the wild-type strain. The data represent the mean and standard deviations of three replicates grown in AB minimal media using glucose as the carbon source. The significance levels, as determined using a one-way ANOVA, of the difference between the wild type and mutant strains are indicated above the bars; ***P < 0.001, no stars indicate no significant difference.

We have previously provided evidence that the production of Bep is regulated by two proteins termed BerA and BerB through a cascade involving two consecutive transcription events that are both activated by c-di-GMP^16^. BerB is an enhancer-binding protein that binds c-di-GMP and activates RpoN-dependent transcription of the *berA* gene coding for a c-di-GMP-responsive transcriptional regulator^15^. An increased level of the BerA protein, in turn, induces the production of Bep^15^. Deletion of *rpfR* results in increased c-di-GMP levels^10^, which could be an underlying cause of increased Bep expression. In this case, one would expect that alteration of cellular c-di-GMP levels by the inactivation of enzymes involved in c-di-GMP metabolism would exert similar effects. A bioinformatics analysis revealed that *B. cenocepacia* H111 codes for 25 putative c-di-GMP modifying proteins; 12 GGDEF domain proteins, 6 EAL domain proteins, and 5 proteins carrying both domains, as well as 2 HD-GYP domain proteins^22^. A collection of *B. cenocepacia* H111 mutant strains with mutations in each of the 25 genes encoding the putative c-di-GMP modifying proteins was recently constructed and characterized^22^. To test whether any of the putative c-di-GMP modifying proteins affect the expression of the *bep* gene cluster in *B. cenocepacia* we generated chromosomal *bepB:*:*lacZ* gene fusions in each of the 25 DGC/PDE mutants. In addition, we also inserted a miniTn7-based *berA-lacZ* fusion^16^ in each of the 25 DGC/PDE mutants. β-galactosidase measurements in AB minimal media using 10 mM glucose as a carbon source revealed that *berA* and *bepB* gene expression was only affected in the *rpfR* mutant (Fig. 4). These results indicate that RpfR regulates Bep production via specific protein interactions and not just high cellular c-di-GMP levels.

### Expression of the *bep* gene cluster depends specifically on RpfR and intracellular c-di-GMP levels

**Fig. 5:**
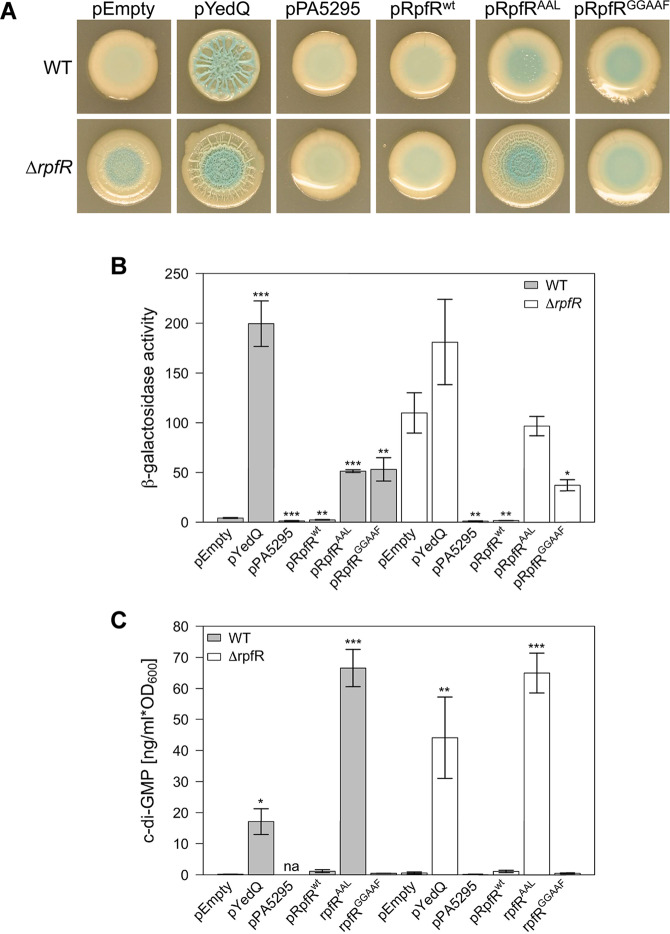
Effect of artificially modified intracellular c-di-GMP levels on Bep expression. Expression vectors for modifying c-di-GMP levels were introduced into *bepB*::*lacZ* reporter strains with wild-type or *rpfR* mutant genetic background. **A** Colony morphology and β-galactosidase expression after growth for 6 days of the indicated *bepB::lacZ* reporter strains on NYG agar plates supplemented with X-gal. **B** Quantification of β-galactosidase activity (Miller units) of the indicated *bepB::lacZ* reporter strains. The values shown are the means ± standard errors of the means (SEM) of 6 measurements (3 independent experiments and 2 technical replicates per strain). **C** Quantification of intracellular c-di-GMP concentrations. The values shown are the means ± SEM of 2 independent experiments. The significance levels, as determined using a one-way ANOVA, of the difference between the strains carrying the empty vector and the strains carrying the indicated expression vectors are indicated above the bars; ***P < 0.001; **P < 0.01; *P < 0.05; no stars indicate no significant difference; na, value below the detection limit.

To elucidate further the role of both RpfR and c-di-GMP in controlling Bep expression, we altered c-di-GMP levels in *B. cenocepacia* by in trans expression of different DGCs and PDEs. We employed the *Escherichia coli* DGC YedQ, the *Pseudomonas aeruginosa* PDE PA5295, the *B. cenocepacia* H111 wild-type RpfR, as well as RpfR variants carrying point mutations in the GGDEF or EAL domain. The expression vectors were conjugated into the wild type and the Δ*rpfR* mutant harboring the *bepB*::*lacZ* fusion, and the resulting strains were assayed for colony morphology and β-galactosidase activity (Fig. 5A). Additionally, *bepB* expression was quantified in liquid cultures (Fig. 5B) and the intracellular c-di-GMP levels were measured (Fig. 5C). The β-galactosidase activities in liquid medium correlated very well with the visual impression of the β-galactosidase activities in macrocolonies, and strains above an expression level of about 60 Miller units showed a wrinkled colony morphology (compare Fig. 5A, B). Heterologous expression of the *E. coli* DGC YedQ massively increased the intracellular c-di-GMP level in both the wild type and the *rpfR* mutant, while expression of the *P. aeruginosa* PDE PA5295 lowered the c-di-GMP level close to the detection limit in both strains (Fig. 5C). RpfR^GGAAF^-expressing strains showed low c-di-GMP levels whereas expression of the RpfR^AAL^ variant increased the c-di-GMP level even above the one observed with the YedQ-expressing strains. This result indicates that despite the fact that RpfR has a net PDE activity in vivo^10,21^, the enzyme can exhibit high DGC activity when the EAL domain is inactive. Given that the EAL domain of RpfR is only activated upon binding of BDSF to the PAS domain of RpfR, it is possible that the DGC activity of RpfR plays an important role when the population density is low and the BDSF QS system has not been triggered. This would be in agreement with a recent report, which suggested that RpfR is mainly a PDE with constrained DGC activity but that in the early growth phase, the DGC activity of RpfR is required to induce expression of polysaccharide genes, causing increased biofilm production^21^. Previous work has shown that BDSF levels rapidly decrease in the stationary phase^23^, and thus starvation could be an alternative trigger for the activation of DGC activity of RpfR.

For the strains with ectopic expression of PA5295 and YedQ, there was a good correlation between intracellular c-di-GMP levels and *bepB* expression. Expression of PA5295 abolished expression of the *bepA-N* cluster and caused a smooth colony morphology in the Δ*rpfR* mutant. On the other hand, the expression of YedQ significantly increased *bepB* expression and induced a wrinkled colony morphology in the wild-type strain. Expression of YedQ in the Δ*rpfR* mutant gave rise to wrinkled colonies, although the increase in *bepB* expression was not significant compared to the vector control strain. However, the two strains showed about a 60-fold difference in intracellular c-di-GMP levels.

In contrast, no clear correlation between intracellular c-di-GMP levels and *bepB* expression or colony morphology was observed when mutated RpfR variants were over-expressed. Although wild-type stains expressing RpfR^AAL^ or RpfR^GGAAF^ showed differences in their intracellular c-di-GMP levels of almost two orders of magnitude, *bepB* expression was almost identical in the two strains (Fig. 5B, C). Interestingly, the RpfR^GGAAF^ variant did not reduce *bepB* expression to the level of the wild type, although the intracellular c-di-GMP level of the strain was very low. This suggests that only when both the GGDEF and the EAL domains are intact, RpfR function as a negative regulator of the *bepA-N* gene cluster.

**Fig. 6:**
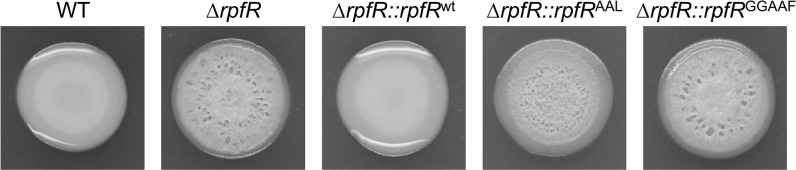
Colony morphology of *B. cenocepacia* H111 strains to express RpfR variants. Alleles for wild-type *rpfR*, *rpfR*^AAL^, and *rpfR*^GGAAF^ were crossed back into the *rpfR* mutant by allelic exchange, generating strains Δ*rpfR::rpfR*^wt^, Δ*rpfR::rpfR*^AAL^, and Δ*rpfR::rpfR*^GGAAF^, respectively. The wild-type strain, the parental strain Δ*rpfR,* and strains encoding RpfR variants were assayed for colony morphology on NYG agar plates.

Overexpression of RpfR variants from a multi-copy plasmid will not only lead to unphysiologically high gene copy numbers per cell but will also result in a mixture of wild-type and mutant RpfR proteins in the wild-type strain. Based on the assumption that RpfR operates not only by modifying the local or global c-di-GMP pool but also through protein-protein interaction, the appropriate stoichiometry with respect to potential interaction partners might be crucial. We, therefore, crossed *rpfR*^*AAL*^ and *rpfR*^*GGAAF*^ alleles into the chromosome, enabling us to express these variants at wild-type levels. Both mutants displayed the wrinkled macrocolony phenotype of the Δ*rpfR* null mutant (Fig. 6), indicating that both the GGDEF and the EAL domain are required for colony wrinkling. It is important to note that this result is different from the experiment shown in Fig. 5A, where the *ΔrpfR* strain was complemented with the *rpfR*^*GGAAF*^ allele on a plasmid. No wrinkling was observed with this strain, suggesting that overexpression of the *rpfR*^*GGAAF*^ variant from a multi-copy plasmid may titrate an interaction partner required for in vivo functioning.

### RpfR variants display DGC as well as PDE activity in vitro

**Fig. 7:**
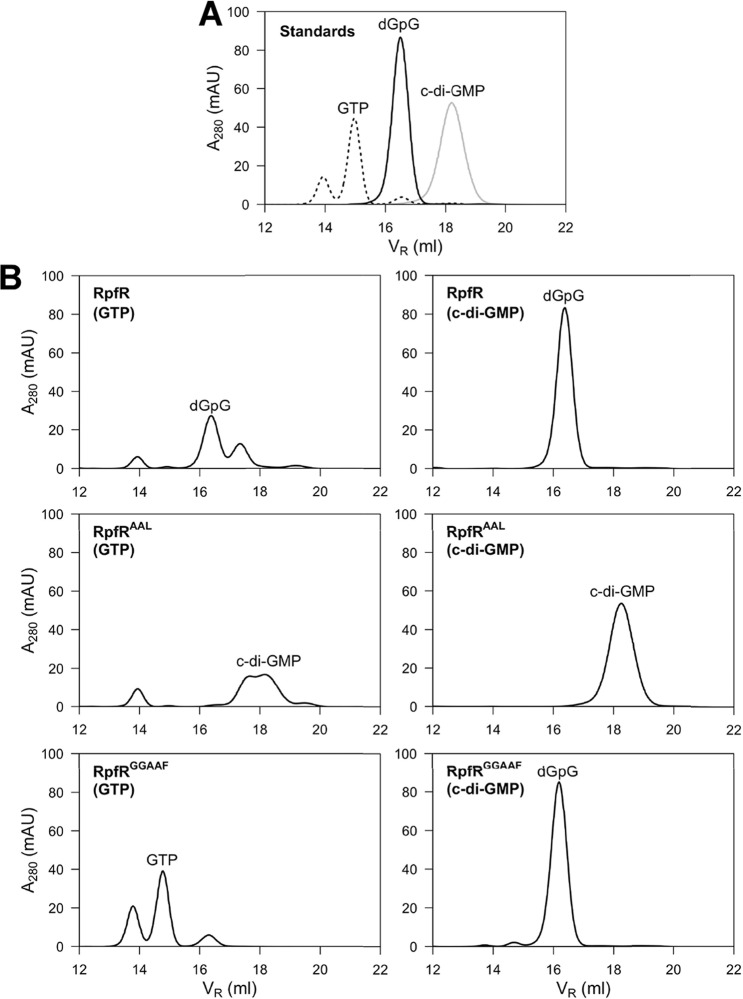
In vitro DGC and PDE activity of RpfR variants. Purified proteins were incubated with GTP or c-di-GMP as substrate and reaction products were analyzed by FPLC. **A** Elution profile of the indicated nucleotide standards. **B** Reaction products of RpfR variants incubated with GTP (left panel) or c-di-GMP (right panel).

A previous study showed that RpfR has net c-di-GMP PDE activity^10^. To biochemically analyze our RpfR variants, we generated expression vectors for RpfR, RpfR^GGAAF^, and RpfR^AAL^, and tested the purified proteins for their ability to metabolize GTP and c-di-GMP (Fig. 7). Wild-type RpfR metabolized GTP as well as c-di-GMP, generating pGpG from both substrates. In contrast, DGC-deficient RpfR^GGAAF^ was only able to degrade c-di-GMP to pGpG and PDE-deficient RpfR^AAL^ was only able to generate c-di-GMP from GTP (Fig. 7). These data confirm that, at least in vitro, both the GGDEF and EAL domain of RpfR are functional and that the created RpfR variants had retained the activity of their respective intact domain.

### The BerB and RpfR proteins physically interact

**Fig. 8:**
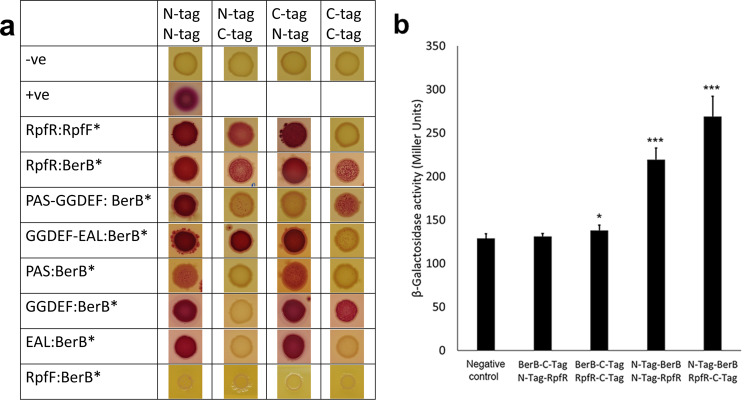
Bacterial two-hybrid assays indicate that RpfR and BerB interact. BerB and RpfF or RpfR or specific domains of RpfR were either N-terminally or C-terminally tagged with one of the two domains of the adenylate cyclase. Pairs of the parental plasmids were used as a negative control, and the Zip domain plasmids described in Karimova 1998^35^ were used as a positive control. **A** *E. coli* harboring the indicated plasmid pairs was grown for 72 h as colonies on MacConkey agar supplemented with 1% maltose before being imaged. Stars (*) are used to discriminate plasmids to indicate if the tested protein was N-terminally tagged (N-tag) or C-terminally tagged (C-tag) with one of the two domains of the adenylate cyclase. Red colonies indicate that the protein fragments physically interact. **B** β-galactosidase assays were used to quantify the strength of the interaction of RpfR and BerB after overnight growth in LB. Error bars indicate the standard deviation of the three independent and two technical repeats used in this assay. The negative control is a pair of the parental plasmids used in the assay. The significance levels, as determined using a one-way ANOVA, of the difference between the strains carrying the indicated expression vectors are indicated above the bars; ***P < 0.001; **P < 0.01; *P < 0.05; no stars indicate no significant difference.

To further investigate whether the specificity of RpfR-regulated Bep expression is due to protein interactions, we employed a bacterial two-hybrid system as detailed in the Methods section. We tested if RpfR could physically interact with the Bep regulators BerA and BerB. While BerA did not bind to RpfR, an interaction between the N-terminal but not the C-terminal region of BerB and RpfR was observed (Fig. 8A). This relatively weak interaction was confirmed by β-galactosidase measurements, which showed a significant increase in activity in the BerB/RpfR pairing (Fig. 8B). Given that recent work has demonstrated that RpfR forms a protein complex with RpfF we also tested whether BerB could interact with RpfF. However, no interaction between BerB and RpfF could be observed. Investigations with shortened versions of RpfR suggested that BerB interacted with both the GGDEF and EAL domain of RpfR while no interaction was observed with the PAS domain of the protein (Fig. 8A). These data suggest that the specificity in the downstream regulatory cascade of RpfR is due to physical interaction with BerB rather than changes of global cellular c-di-GMP levels. The specificity of the interaction of BerB to the GGDEF and EAL domain of RpfR also suggests that c-di-GMP might change the affinity of the interaction of BerB and RpfR.

## Discussion

In recent years, c-di-GMP has emerged as an important bacterial second messenger and as a key player in a wide range of cellular processes such as biofilm formation, virulence, motility, and cell cycle. A number of c-di-GMP effector proteins have been identified that control the transcription of target genes. However, very little is known about how these regulators specifically control certain phenotypes in response to changes in intracellular c-di-GMP levels. The study of c-di-GMP-dependent regulatory systems is further complicated by the fact that organisms often encode dozens of apparently redundant enzymes that synthesize and hydrolyze c-di-GMP. *B. cenocepacia* H111, for example, encodes 25 such proteins. The mode of action of enzymes involved in c-di-GMP metabolism is not limited to the ‘making and breaking’ of the signal molecule but many of these enzymes also comprise signal-sensing domains, protein interaction domains, or localization signals^24,25^.

In a previous study, we found a substantial overlap of the CepIR and the RpfFR regulons in *B. cenocepacia* H111, however, we also identified genes that were almost exclusively regulated by one of the two QS systems^12^. The *aidA* gene, for example, which is required for the killing of the nematode *Caenorhabditis elegans*, harbors a *cep* box in its promoter region and is stringently regulated by AHL. Expression of the lectin BclB, on the other hand, is strongly dependent on BDSF^12^. In contrast, expression of the large surface protein BapA requires both signaling molecules. Taken together, our data suggested that the two QS systems operate in parallel and that an unknown c-di-GMP effector either directly activates the transcription of target genes or that the two cascades converge and control the expression of a common unknown regulator^12^. We were quite intrigued by the question of how such signaling specificity is achieved and were interested in identifying the c-di-GMP effector downstream of RpfR.

The observation that inactivation of the RpfFR QS system leads to wrinkled macrocolony morphology provided us with a convenient phenotypic assay as a readout for RpfFR activity. We first identified the extracellular matrix component responsible for the distinct three-dimensional structure of Δ*rpfR* macrocolonies. We show that the water-insoluble EPS Bep^17^ is responsible for the wrinkled morphotype of Δ*rpfR* macrocolonies and that cepacian is not essential for wrinkling but does affect the architecture of the macrocolony (Fig. 2). Bep expression was previously shown to be regulated by the c-di-GMP effectors BerA and BerB^14–16^ and we show here that deletion of *berA* or *berB* is sufficient to abolish the wrinkled macrocolony morphology of Δ*rpfR*, suggesting that RpfR represses Bep production through modulating BerA or BerB expression or activity. Our data suggest that this regulation occurs through the direct interaction of RpfR with BerB, which, along with the sigma factor RpoN, is responsible for *berA* transcription^16^. However, subcellular localization experiments with fluorescently tagged BerB and RpfR proteins provided no evidence for a co-localization of the two proteins, suggesting that the interaction may only be transient (data not shown). This is also supported by two-hybrid interaction experiments, which suggested that the interaction between RpfR and BerB is relatively weak when compared to a control (Fig. 8). RpfR and RpfF have been shown to form a heterohexameric protein complex consisting of three RpfF and three RpfR monomers^11^. However, we could not observe an interaction between RpfF and BerB, indicating that RpfR is the only interaction partner in this protein complex.

In a previously published genome-wide transcriptome analysis, we showed that about 25% of the top-ranked differentially expressed genes were up-regulated in a *rpfF* mutant^12^. Among the down-regulated genes were the two cepacian gene clusters *bce-I* and *bce-II*. Although the inactivation of cepacian production in the Δ*rpfR* background (Δ*rpfR* Δ*bceC*, Δ*gtaB)* did not abolish the wrinkled phenotype, it led to flatter and smoother macrocolonies (Fig. 2B), suggesting that cepacian production contributes to the architecture of the macrocolonies but is not the actual cause of wrinkling. It is worth noting that the type of EPS produced depends on the medium and the surface used^12^ (Supplementary Fig. 1 and 3), and it is therefore not surprising that in the global gene expression studies, which were performed in liquid LB medium, only cepacian but not the Bep EPS cluster was identified as part of the BDSF regulon^12^. This hypothesis is further supported by a recent transcriptome study that showed that RpfR negatively controls *bep* gene expression in cells grown as a biofilm on polystyrene beads while no significant effect was observed on the expression of *bce* genes^21^. Another important finding of our study is that the expression of the bep operon is not homogenous across the macrocolony but appears to be constrained to certain regions of the colony. The finding that *bep* expression was often found to be particularly high within the ridges of the wrinkled colony (Fig. 5A; Supplementary Fig. 2) suggests that localized expression of Bep EPS of a subpopulation of cells is essential for macrocolony wrinkling. Additional work will be required to investigate the interplay of Bep and cepacian in biofilm structural development and to determine their spatial expression patterns in different biofilm models.

While genomic analyses have revealed that GGDEF and EAL domains frequently occur in tandem as part of multidomain proteins, often one of the two domains is catalytically inactive and only a few examples of truly bifunctional DGCs/PDEs have been described to date^24,25^. In this context, the BDSF sensor RpfR is particularly interesting since it is a bifunctional GGDEF-EAL protein that links QS with c-di-GMP turnover. Previous work has shown that RpfR has a net PDE activity in vivo^10,21^ and thus can be considered a c-di-GMP sink. By contrast, *bep* gene expression is stimulated by high c-di-GMP levels, as BerB needs to bind c-di-GMP to activate the transcription of downstream genes. Here, we provide evidence that the BDSF receptor is indeed capable of both synthesizing and hydrolyzing c-di-GMP in vitro and that both the EAL and GGDEF domains must be intact for full functionality of the RpfFR QS system in vivo. This result is in line with a recent study showing that in evolution experiments, mutations in both the GGDEF and EAL domain can provide a fitness benefit in a biofilm bead model system^21^.

**Fig. 9:**
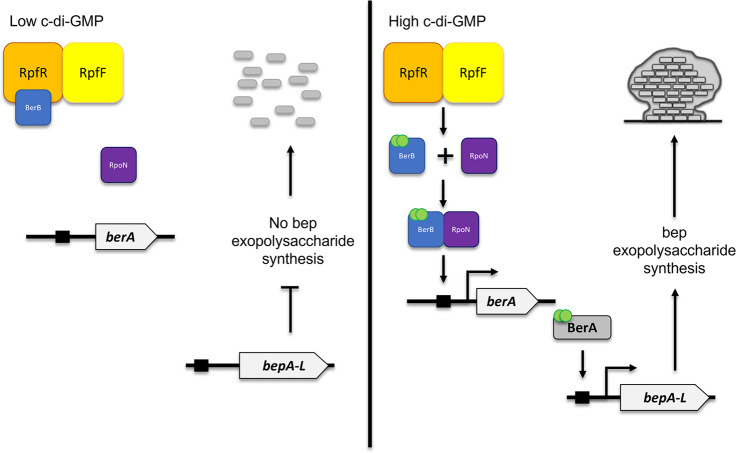
Model of RpfR regulation of Bep exopolysaccharide synthesis. BerB binds to RpfR in complex with RpfF preventing its localization to the *berA* promoter. Rising levels of c-di-GMP lead to the binding of c-di-GMP to BerB, inhibiting the protein interaction and allowing the released BerB to stimulate transcription of *berA*, and the BerA protein then activates transcription of the *bep* operon. Modified from Fazli et al., 2017^16^.

In *E. coli*, two c-di-GMP modules regulate the transcription of CsgD, a key biofilm regulator of amyloid curli fiber production. The PDE YciR, which displays 54% amino acid identity to RpfR, was demonstrated to act as a bi-functional trigger enzyme that connects the two control modules: on the one hand, YciR degrades c-di-GMP generated by a module I and, on the other hand, it inhibits the YdaM DGC through direct protein-protein interaction^26^. Such a signaling cascade involving a trigger enzyme, which in addition to its enzymatic function also acts through physical interaction with other proteins, demonstrates how spatial sequestration and, therefore, specificity of c-di-GMP signaling can be achieved within a cell^26,27^. While both YciR and RpfR are composite proteins containing a GGDEF as well as an EAL domain, DGC activity of YciR appears to be weak and was shown not to be required for expression of the CsgD target gene *csgB*^26^. In the case of RpfR, however, both the GGDEF and the EAL had to be catalytically functional in order to restore the wild-type colony morphology (Fig. 6). Based on the homology of YciR and RpfR, it is tempting to speculate that RpfR acts in a similar way as YciR, albeit in conjunction with different interaction partners since no homologs to the interaction partners of YciR could be identified in the *B. cenocepacia* H111 genome. On the basis of our data, we propose a model in which RpfR inhibits the activity of BerB through protein-protein interaction (Fig. 9). At low c-di-GMP levels, RpfR binds BerB to sequester this RpoN-dependent activator to the RpfR/RpfF complex such that BerB cannot activate expression of BerA and thus Bep biosynthesis is not induced. This binding appears to be transient and as the c-di-GMP levels rise, possibly by the enzymatic action of RpfR, BerB is released from the RpfR/RpfF complex and associates with RpoN to activate the transcription of BerA. Using surface plasmon resonance assays, it was shown that BerB can bind c-di-GMP. Moreover, electrophoretic mobility assays showed that BerB binds to the *berA* promoter and that DNA-binding was not stimulated by c-di-GMP^16^. Given that a *rpfR* null mutant displays a wrinkled macrocolony morphology, we hypothesize that BerB can associate with c-di-GMP molecules from the cellular pool to activate *bep* gene expression. In the wild-type strain, the RpfR/RpfF complex sequesters BerB and the PDE activity of the EAL domain of RpfR may prevent the binding of c-di-GMP to BerB. In agreement with our data, mutation of the EAL domain will allow BerB to bind c-di-GMP, which as a consequence, may result in the release of BerB/c-di-GMP from the complex. Whether the GGDEF domain is involved in loading BerB with c-di-GMP or whether it is a docking site for BerB is an interesting question for future research.

## Methods

### Bacterial strains and growth conditions

All strains used in this study are listed in Table 1. Unless otherwise stated, *E. coli* and *B. cenocepacia* H111 strains were routinely cultured aerobically in Luria–Bertoni (LB) Lennox broth (Difco) medium at 37 °C. Culture media were solidified with 1.5% (w/v) agar and supplemented with the following antibiotics where appropriate: ampicillin, 100 μg/ml; chloramphenicol, 20 μg/ml; gentamycin, 20 μg/ml; kanamycin, 50 μg/ml; and trimethoprim, 50 μg/ml.

**Table 1:** Bacterial strains used in this study.

Strains	Characteristics	Source/Reference
*Burkholderia cenocepacia*		
H111	Clinical isolate from CF patient	^3^
∆*rpfF*	H111 with in-frame deletion of *rpfF*	This study
∆*rpfR*	H111 with in-frame deletion of *rpfR*	This study
∆*rpfF* ∆*rpfR*	H111 with in-frame deletion of *rpfF* and *rpfR*	This study
∆*bceC*	H111 with in-frame deletion of *bceC*	This study
∆*gtaB*	H111 with in-frame deletion of *gtaB*	This study
∆*bepB*	H111 with in-frame deletion of *bepB*	This study
∆*berB*	H111 with in-frame deletion of *berB*	^16^
∆*berA*	H111 with in-frame deletion of *berA*	This study
∆*bceC* ∆*gtaB*	H111 with in-frame deletion of *bceC* and *gtaB*	This study
∆*rpfR* ∆*bceC*	H111 with in-frame deletion of *rpfR* and *bceC*	This study
∆*rpfR* ∆*gtaB*	H111 with in-frame deletion of *rpfR* and *gtaB*	This study
∆*rpfR* ∆*bepB*	H111 with in-frame deletion of *rpfR* and *bepB*	This study
∆*rpfR* ∆*berB*	H111 with in-frame deletion of *rpfR* and *berB*	This study
∆*rpfR* ∆*berA*	H111 with in-frame deletion of *rpfR* and *berA*	This study
∆*rpfR* ∆*bceC* ∆*gtaB*	H111 with in-frame deletion of *rpfR, bceC* and *gtaB*	This study
∆*cdpA*	H111 with in-frame deletion of *cdpA*	^22^
*∆bcal0430*	H111 within-frame deletion of *bcal0430*	^22^
*∆bcal1635*	H111 within-frame deletion of *bcal1635*	^22^
*∆bcal1975*	H111 within-frame deletion of *bcal1975*	^22^
*∆bcal2852*	H111 within-frame deletion of *bcal2852*	^22^
*∆bcam0748*	H111 within-frame deletion of *bcam0748*	^22^
*∆bcam1161*	H111 within-frame deletion of *bcam1161*	^22^
*∆bcam1554*	H111 within-frame deletion of *bcam1554*	^22^
*∆bcam1670*	H111 within-frame deletion of *bcam1670*	^22^
*∆bcam2256*	H111 within-frame deletion of *bcam2256*	^22^
*∆bcam2822*	H111 within-frame deletion of *bcam2822*	^22^
*∆bcam2836*	H111 within-frame deletion of *bcam2836*	^22^
*∆bcas0398*	H111 within-frame deletion of *bcas0398*	^22^
*∆bcal0621*	H111 within-frame deletion of *bcal0621*	^22^
*∆bcal2449*	H111 within-frame deletion of *bcal2449*	^22^
*∆bcam1160*	H111 within-frame deletion of *bcam1160*	^22^
*∆bcal0652*	H111 within-frame deletion of *bcal0652*	^22^
*∆bcal1100*	H111 within-frame deletion of *bcal1100*	^22^
*∆bcal2749*	H111 within-frame deletion of *bcal2749*	^22^
*∆bcal3188*	H111 within-frame deletion of *bcal3188*	^22^
*∆bcam0158*	H111 within-frame deletion of *bcam0158*	^22^
*∆bcam2426*	H111 within-frame deletion of *bcam2426*	^22^
*∆bcas0263*	H111 within-frame deletion of *bcas0263*	^22^
*∆bcas0378*	H111 within-frame deletion of *bcas0378*	^22^
*bepB*::*lacZ*	H111 wild type with *lacZ* inserted downstream of *bepB*	This study
∆*rpfR bepB*::*lacZ*	H111 ∆*rpfR* with *lacZ* inserted downstream of *bepB*	This study
∆*berA bepB*::*lacZ*	H111 ∆*berA* with *lacZ* inserted downstream of *bepB*	This study
∆*rpfR::rpfR*^*wt*^	H111 ∆*rpfR* reverted back to wild-type genotype	This study
∆*rpfR::rpfR*^*GGAAF*^	H111 carrying *rpfR* allele with mutated GGDEF domain	This study
∆*rpfR::rpfR*^*AAL*^	H111 carrying *rpfR* allele with mutated EAL domain	This study
*Escherichia coli*		
DH5α	Strain for standard cloning applications	Laboratory stock
Top10	Strain for standard cloning applications	Laboratory stock
*E. coli* M15[pREP4]	Expression host for recombinant proteins	Qiagen
SY327	Maintenance of pGPI-SceI based vectors	^18^
DMH1	Bacterial two-hybrid strain	^25^

### Transformation of *E. coli* and *B. cenocepacia H111* strains

*E. coli* strains were transformed by standard electroporation procedures or the classical CaCl_2_ method. *B. cenocepacia* H111 strains were transformed by tri-parental mating^28^. Briefly, donor, helper and recipient strains were grown overnight in LB at 37 °C with shaking. Two ml of each strain were harvested, washed, and resuspended in 500 μl (donor and helper) or 1 ml (recipient) LB Lennox broth. Donor and helper cells (100 μl each) were mixed and incubated for 20 min at room temperature. Recipient cells (200 μl) were added and the mixture was spot-inoculated onto the surface of prewarmed LB agar plates. After incubation at 37 °C for 6 h, cells were scraped off, resuspended in 500 μl 0.9% NaCl and plated on Pseudomonas Isolation Agar (Difco) supplemented with the appropriate antibiotics for counter-selection.

### DNA manipulation

Plasmid DNA was isolated with the QIAprep Spin Miniprep Kit (Qiagen), chromosomal DNA was prepared with the DNeasy Blood & Tissue Kit (Qiagen), and DNA fragments were purified using the Zymoclean Gel DNA Recovery Kit (Zymo Research), where required. For cloning purposes, DNA was amplified with Phusion High-Fidelity DNA Polymerase (New England Biolabs), to confirm vector constructs and mutants, GoTaq DNA Polymerase (Promega) was employed. Oligonucleotides used in this study are listed in Supplementary Table 1.

### Construction of expression vectors

Plasmids employed in this study are listed in Supplementary Table 2. Coding sequences for *rpfR*, *rpfR*^*GGAAF*^, *rpfR*^*AAL*^, *berA*, and *yedQ* were subcloned into the broad-host-range cloning vector pBBR1MCS-5^29^ as follows: *rpfR*, *rpfR*^*GGAAF*^, and *rpfR*^*AAL*^ as XbaI-HindIII fragments from pBBR-rpfR, pBBR-rpfR^GGAAF^, and pBBR-rpfR^AAL^, respectively, generating pRpfR^wt^, pRpfR^GGAAF^, and pRpfR^AAL^; *berA* as a BamHI-XbaI fragment from pBBR2-Bcam1349, generating pBerA; and *yedQ* as a HindIII-BamHI fragment from pYhck, generating pYedQ. To generate pRpoN and pBerB, coding sequences together with the putative promoter region were PCR-amplified from genomic DNA using primer pairs P103/P104 and P280/P281, respectively, and cloned as XbaI-HindIII fragments into pBBR1MCS-5.

For heterologous expression, the *rpfR* gene was PCR-amplified using primers pQE-rpfR-F and pQE-rpfR-R, digested with BamHI and HindIII and cloned into pQE-32 digested with the same enzymes, giving rise to plasmid pQE-RpfR, which was transformed into *E. coli* M15[pREP4]. Point mutations were inserted into pQE32-rpfR by site-directed mutagenesis, using the QuikChange site directed mutagenesis system (Agilent)^10^, generating plasmids pQE-RpfR^GGAAF^ and pQE-RpfR^AAL^.

### Construction of *B. cenocepacia* H111 mutants

To generate unmarked gene deletions or introduce *rpfR* variants into the chromosome, we used the method described by Flannagan et al.^28^, which is based on the homing nuclease I-SceI and allows for markerless gene deletion (knock out) as well as gene insertion (knock-in). Since *B. cenocepacia* H111 is not very sensitive to tetracycline, the XhoI-SalI fragment of pDAI-SceI containing the *tetA* and *tetR* genes was replaced with the PCR-amplified (primers GmR_F and GmR_R) gentamicin-3-acetyltransferase gene from pBBR1MCS-5, generating the I-SceI expression plasmid pDAIGm-SceI.

Knock-out plasmids were generated by PCR amplification of the flanking regions of target genes (~1200 bp for *rpfR*, ~600 bp for all other target genes) from genomic DNA of *B. cenocepacia* H111, incorporating EcoRI/NcoI and NcoI/KpnI recognition sites into left and RHAs, respectively, using the following primer pairs: P14/P15 and P16/P20 for pGPI_∆rpfR; P47/P48b and P49/P50b for pGPI_∆rpfF; P46/P47 and P14/P15 for pGPI_∆rpfFR; P66/P67 and P68/P69 for pGPI_∆bepB; P73/P74 and P75/P76 for pGPI_∆bceC; P80/P81 and P82/P83 for pGPI_∆gtaB; P86/87 and P88/89 for pGPI-ΔrpoN; P93/P94 and P95/P96 for pGPI_∆berA. PCR fragments were digested with the respective enzymes and cloned into the EcoRI/KpnI-linearized pGPI-SceI plasmid in a three-way ligation, thereby joining the two homology arms through the NcoI site. All knock-out plasmids were verified by sequencing.

The multiple cloning site of plasmid pGPI-SceI was modified by the insertion of annealed oligonucleotides MCS-GPI_F and MCS-GPI_R into the EcoRI/XbaI-linearized vector, generating pGPI-SceI-ΔXbaI. The unique PstI site in the vector backbone was removed by linearizing pGPI-SceI-ΔXbaI with PstI, blunting ends with T4 DNA polymerase and re-ligation, giving rise to pGPI-SceI-ΔXbaI-ΔPstI. Annealed oligonucleotides MCS2-GPI_F and MCS2-GPI_R were ligated into the NcoI/KpnI-linearized pGPI-SceI-ΔXbaI-ΔPstI vector, thus generating plasmid pGPI2-SceI, which carries unique restriction sites for EcoRI, BglII, EcoRV, NcoI, SpeI, XbaI, XhoI, SphI, KpnI in its multiple cloning site.

To introduce point mutations into the GGDEF and EAL domain of chromosomally encoded RpfR, the respective *rpfR* variants were excised as XbaI-KpnI fragments from plasmids pRpfR^GGAAF^, pRpfR^AAL^, and pRpfR^wt^ and subcloned into pGPI2-SceI cleaved with the same enzymes, yielding plasmids pGPI-rpfR^GGAAF^, pGPI-rpfR^AAL^, and pGPI-rpfR^WT^. All knock-in plasmids were confirmed by sequencing.

*B. cenocepacia* H111 locus tags, orthologs in the closely related *B. cenocepacia* J2315, and products of genes investigated in this study are summarized in Table 2.

**Table 2:** Mutants constructed in this study.

Locus Tag^a^	Ortholog in J2315^b^	Product description^c^	Gene/cluster name
I35_2858	BCAL0813	RNA polymerase factor sigma-54	*rpoN*
I35_4474	BCAM0580	Cis-2-dodecenoic acid receptor RpfR	*rpfR*
I35_4475	BCAM0581	BDSF synthase rpfF	*rpfF*
I35_4769	BCAM0855	UDP-glucose dehydrogenase	*bceC*
I35_4929	BCAM1010	UTP--glucose-1-phosphate uridylyltransferase	*gtaB*
I35_5181	BCAM1331	Tyrosine-protein kinase Wzc	*bepB*
I35_5193	BCAM1342	Sigma-54 dependent transcriptional regulator	*berB*
I35_5200	BCAM1349	CRP/FNR family transcriptional regulator (10)	*berA*
I35_4767-I35_4777	BCAM0854-BCAM0864	Cepacian	*bce-I*
I35_4922-I35_4930	BCAM1003-BCAM1011	Cepacian	*bce-II*
I35_5180-I35_5191	BCAM1330-BCAM1341	*Burkholderia* exopolysaccharide	*bepA-N*

^a^Nomenclature according to GenBank accession numbers HG938370, HG938371, and HG938372.

^b^Orthologs in *B. cenocepacia* J2315 were identified by BLAST search.

^c^Product description according to GenBank accession numbers HG938370, HG938371, and HG938372.

### Construction of the *bepB*-*lacZ* reporter strains

To generate pGPI-bepB::lacZ, a pGPI-SceI based plasmid to introduce the *lacZ* gene along with its ribosome binding site downstream of the *bepB* gene, left and right homology arms (RHAs) were amplified from genomic DNA of *B. cenocepacia* H111. The RHA was PCR-amplified using primer pair P69/P122, the left homology arm (LHA) was amplified with primer pair P120/P121. The XbaI/KpnI-digested RHA was first cloned into pBluescript SK(+) linearized with the same enzymes, resulting in pBluescript-RHA. The EcoRI/PstI-digested LHA was cloned into pSUP3535 linearized with the same enzymes, giving rise to pSUP3535-LHA-lacZ. The KpnI-XbaI fragment of pBluescript-RHA comprising the RHA, and the EcoRI-XbaI fragment from pSUP3535-LHA-lacZ, comprising the LHA and the *lacZ* gene, were cloned into the EcoRI/KpnI-linearized pGPI-SceI plasmid, generating pGPI-bepB::lacZ.

### Macrocolony morphology and cepacian production assays

Strains were precultured overnight in LB Lennox broth under aeration at 37 °C. To assay macrocolony morphology, 3 μl of precultures were spotted on NYG agar plates (0.5% peptone, 0.3% yeast extract, 2% (w/v) glycerol, and 1.5% agar) or AB minimal medium^30^ supplemented with 10 mM of glucose or 1.5% (w/v) glycerol as carbon source and incubated for 3 days at 37 °C, followed by at least three days at room temperature. Cepacian production was assayed by streaking precultures on YEM agar plates (0.05% yeast extract, 0.4% mannitol, and 1.5% agar) and incubation at 37 °C for 2 days. All strains to be compared were grown in parallel on single square Petri dishes (120 mm, Greiner Bio-One).

### Determination of β-galactosidase activity

To assay β-galactosidase activity within macrocolonies, NYG agar plates supplemented with 5-bromo-4-chloro-3-indolyl-β-D-galactopyranoside (X-Gal; 40 μg/ml) were spot inoculated as described above. For quantification of β-galactosidase activity, strains to be tested were grown overnight in LB Lennox broth at 37 °C and used to inoculate 100-ml Erlenmeyer flasks containing 20 ml of, unless otherwise stated, AB minimal medium^30^ supplemented with 10 mM of glucose or 1.5% (w/v) glycerol as a carbon source to an OD_600_ of 0.05. Cultures were incubated with agitation (220 rpm) for 24 h at 37 °C. β-galactosidase activity was quantified as described by Stachel et al.^31^ with slight modifications. Briefly, 50–200 μl of cell culture were harvested and resuspended in a 500 μl Z-buffer. After addition of 25 μl of CHCl_3_ and 25 μl of 0.05% SDS, the cell suspension was vortexed for 10 seconds and then incubated at 30 °C for 15 min. The reaction was started by adding 200 μl of *o*-nitrophenyl-β-d-galactopyranosid (ONPG; 4 mg/ml) and incubating at 30 °C. The reaction was stopped by the addition of 250 μl of 1 M Na_2_CO_3_. Cell debris was removed by centrifugation, and absorbance was recorded at 420 nm and 550 nm. β-galactosidase activity was calculated as Miller Units, using the formula Miller Units = 1000 × (OD_420_ − (1.75 × OD_550_))/(time[min] × [ml] × OD_600_).

### DGC/PDE assays

To assess the enzymatic activity of RpfR, a DGC/PDE assay^32^ was performed using 2 μM of purified RpfR or its variants resuspended in 50 mM Tris-Cl pH 7.8, 500 mM NaCl, 2 mM MgCl_2_. The reaction was started by adding GTP (Sigma) or c-di-GMP (Biolog) as a substrate to a final concentration of 100 μM and incubating the reaction mix at 37 °C. After 180 min of incubation, the protein was denatured by heating to 99 °C for 5 min, followed by centrifugation at 20,000*g* for 15 min. Supernatants were analyzed on an Äkta FPLC system, using 1 ml ResourceQ columns (GE Healthcare) and a linear gradient from 0.5 to 100% of 1 M NH_4_HCO_3,_ pH 8. GTP, c-di-GMP, and pGpG (Biolog) were used as standards at a concentration of 100 μM.

### Extraction and quantification of c-di-GMP

To quantify intracellular c-di-GMP levels, strains to be analyzed were grown in LB Lennox broth to an OD_600_ of ~1.8 at 37 °C with aeration. Extraction and quantification of c-di-GMP was performed as described by Burhenne and Kaever^33^.

### Bioassay for BDSF production

To test H111 strains for BDSF production, cross-streak experiments with the H111-rpfF_Bc_/pAN-L15 biosensor strain were performed^34^.

### Bacterial two-hybrid analysis

For bacterial two-hybrid analysis, we used the system described by Karimova, 1998^35^ using a split adenylate cyclase. Primer pairs P301/P302 and P303/304 were used to PCR amplify the *berB*. The resulting PCR fragments and the plasmids pUT18 and pUT18C were then digested with the restriction enzymes BamHI and HindIII before being ligated together to create pUT18-BerB and pUT18C-BerB. The following primer pairs were used to create PCR fragments of the full-length *rpfF* gene, full-length *rpfR* gene, and specific domains of the *rpfR* gene: P272/P273 for full length *rpfF*; P214/P215 for full-length *rpfR*; P214/P238 for *rpfR* PAS-GGDEF; P237/P215 for *rpfR* GGDEF-EAL; P214/P236 for *rpfR* PAS; P237/P238 for *rpfR* GGDEF; and P239/P215 for *rpfR* EAL. The resulting PCR fragments were digested with the restriction enzymes XbaI and KpnI alongside the plasmids pKT25 and pKNT25. Then each fragment was ligated into each plasmid to create pKT25-RpfF, pKNT25-RpfF, pKT25-RpfR, and pKNT25-RpfR and the various derivatives. The plasmids were introduced into *E. coli* DHM1 by electroporation and selected for on first single selection plates and then double selection plates to create DHM1 strains containing one pUT18-derivative or pUT18C-derivative with one pKT25-derivative or pKNT25-derivative. Pairs of the parental plasmids were used as a negative control, and a Zip domain plasmid pair^35^ was used as a positive control. Five microlitres of the DHM1 strains were grown on MacConkey agar supplemented with 1% maltose at 30 °C for 72 h before being imaged.

### Determining the β-galactosidase activity of the bacterial two-hybrid mutants

DMH1 strains were grown overnight at 30 °C in LB cultures with the appropriate antibiotics before inoculating 100 µl of this culture into agitated conical flasks (220 rpm) containing 10 ml LB. Cultures were grown overnight at 30 °C and 200 µl samples were used for β-galactosidase assays after permeabilization of the cells with chloroform-sodium dodecyl sulfate^36^.

## Supplementary information

Supplementary Material

## Data Availability

The data underlying this article are available within the article and the accompanying Supplementary Information file. Additional data are available from the corresponding authors upon request.
