# Supplementary material for: The BDSF quorum sensing receptor RpfR regulates Bep exopolysaccharide synthesis in Burkholderia cenocepacia via interaction with the transcriptional regulator BerB
Source: NPJ Biofilms Microbiomes. 2022 Nov 22;8:93. doi: 10.1038/s41522-022-00356-2 (PMC9684580; doi:10.1038/s41522-022-00356-2)
Supplement: Supplementary file 1 — Supplementary Material [file 41522_2022_356_MOESM1_ESM.pdf]

Supplementary Material

Expression of the *Burkholderia cenocepacia* Bep exopolysaccharide is under negative control of the BDSF-dependent quorum sensing system

Elisabeth Steiner[a], Rebecca E. Shilling[a], Anja M. Richter[b], Nadine Schmid[a], Mustafa Fazli[b], Volkhard Kaever[c], Urs Jenal[d], Tim Tolker-Nielsen[b#] and Leo Eberl[a#]

[a]Department of Microbiology, University of Zürich, Zürich, Switzerland; [b]Department of Immunology and Microbiology, Faculty of Health Sciences, University of Copenhagen, Copenhagen, Denmark; [c]Research Core Unit Metabolomics, Hannover Medical School, Hannover, Germany; [d]Focal Area of Infection Biology, Biozentrum, University of Basel, Basel, Switzerland

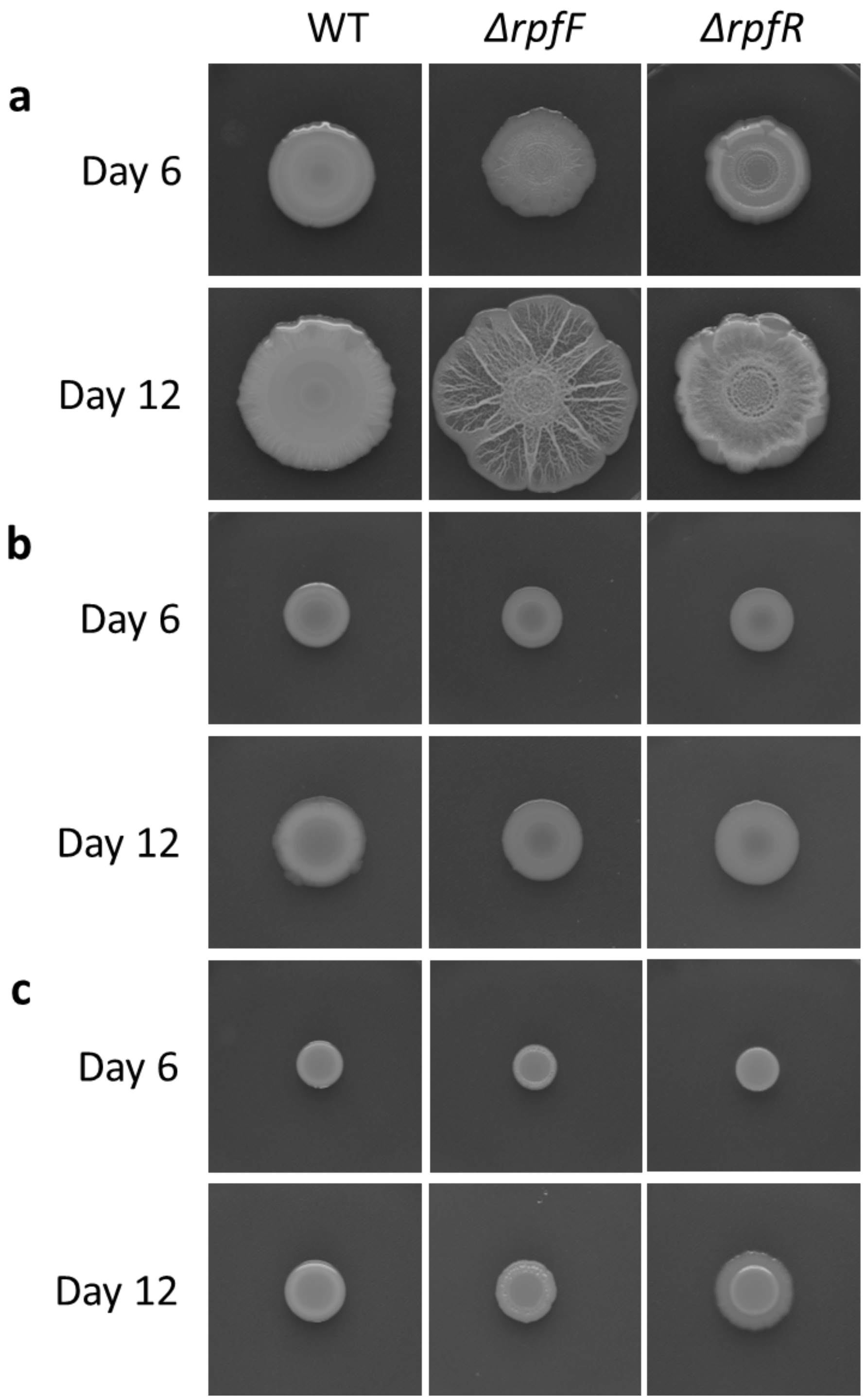

Supplementary Figure 1. Colony morphology of *B. cenocepacia* strains on NYG, AB 1.5% glycerol or AB glucose 10mM. Cell suspensions of wild-type *B. cenocepacia* H111 (WT) and mutants carrying an in-frame deletion of *rpfF*, *rpfR* or both genes were spotted on NYG or AB minimal media with 1.5% glycerol or 10mM glucose as the carbon source agar plates. Colony morphology was assayed after growth for 6 and 12 days.

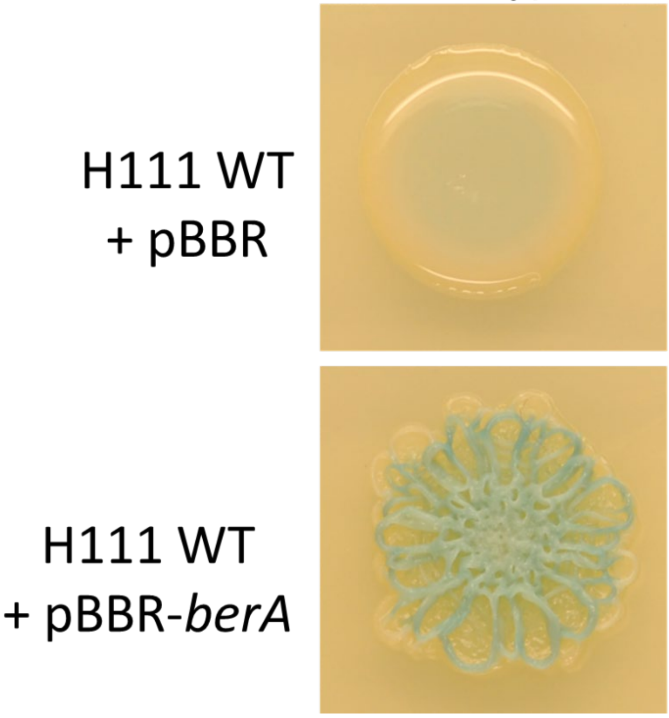

Supplementary Figure. 2. Macrocolony morphology and localized β-galactosidase expression of the *bepB*::*lacZ* reporter strains with and without ectopic expression of *berA* after growth for 12 days on NYG agar plates supplemented with X-gal and appropriate antibiotics.

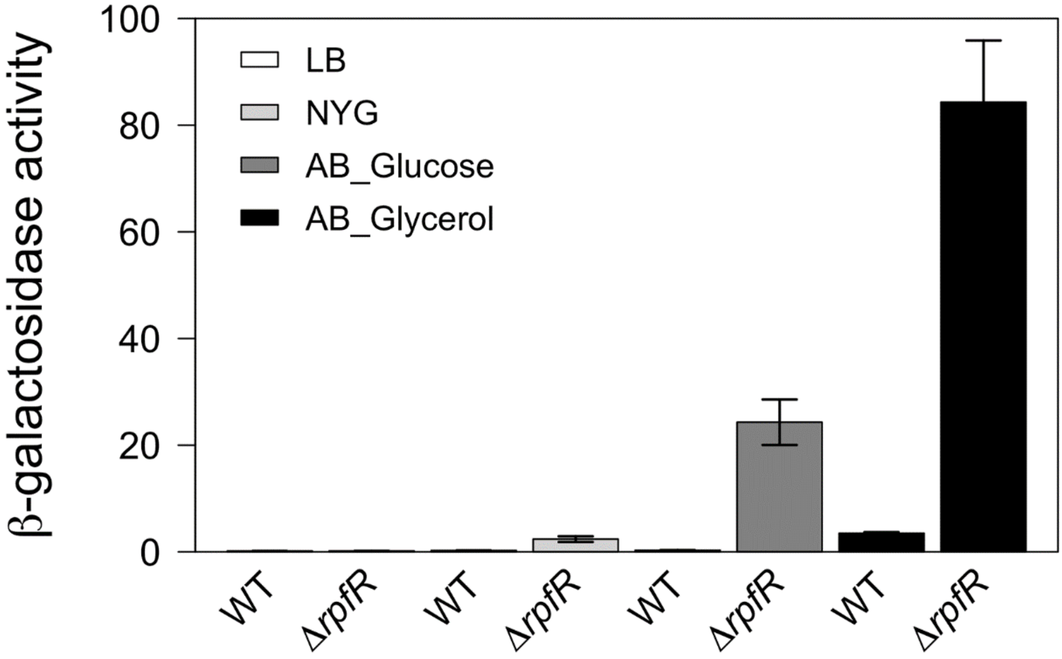

Supplementary Figure. 3. Influence of growth media on Bep expression. Quantification of β-galactosidase activity (Miller units) of strains harbouring the *bepB*::*lacZ* reporter in the wild-type (WT) and *rpfR* mutant (ΔrpfR) background. Reporter strains were grown for 24 h in LB Lennox broth (LB), NYG medium (NYG), AB minimal medium supplemented with 1 % glucose (AB_Glucose), and AB minimal medium supplemented with 1.5 % glycerol (AB_Glycerol).

Supplementary Table 1: Oligonucleotides used in this study

Name	Sequence (5'-3')	Restriction site
GmR_F	ACTGCTCGAGCTAACTCACATTAATTGC	*Xho*I
GmR_R	ACTGGTCGACTTGTTAGGTGGCGGTAC	*Sal*I
MCS_GPI_F	AATTCAGATCTGATATCCATGGCTCGAGCATGCGGTAC	
MCS_GPI_R	CTAGGTACCGCATGCTCGAGCCATGGATATCAGATCTG	
MCS2_GPI_F	CATGGACTAGTTCTAGACTCGAGCATGCGGTAC	
MCS2_GPI_R	CGCATGCTCGAGTCTAGAACTAGTC	
pQE-rpfR-F	GGATCCATATGGATGACGAAAACGATAGCGCG	
pQE-rpfR-R	AAGCTTTCAGGCGATCAGCCTGAGCT	
P14	GTACGAATTCCGCACGCTGGTCGGCGACGAC	
P15	ACTGAGATCTCCATGGACACGCCCCGATCCAG	
P16	ACTGGGATCCATGGCCTGAGCGTCGTTCCGGAC	
P20	TCGAAAGCTTGATCGGCGTCCTGACGTCG	
P46	TGACGGTACCACGGTGTAGCCGAAGTAG	
P47	AGTCCCATGGTATGTCCTCGTGAG	
P48b	GACTGAATTCTCATGCCGATCGGTGAC	
P49	CTGACCATGGGCACGGTGTAATGCGAC	
P50b	TGACGGTACCGAACATCGTGCATCAGTTC	
P66	AGTCGAATTCCCGCATGGATCATGTTG	*EcoR*I
P67	CAGTCCATGGGCAACTGGACCAGCGTG	*Nco*I
P68	AGTCCCATGGCCTGATGTCGCTTGCATG	*Nco*I
P69	GTCAGGTACCGGTCGAATGCAACG	*Kpn*I
P73	TGACGGTACCGAAGTTCGTGTTCGACC	*Kpn*I
P74	AGTCCCATGGTGTGTCGGACCCCTTGAC	*Nco*I
P75	AGTGCCATGGCGTAACCCGTTTTCCGTC	*Nco*I
P76	CTGAGAATTCACCACTTTGGCGTCG	*EcoR*I
P80	AGTCGGTACCGGAAATATCGGTATTC	*Kpn*I
P81	GTCACCATGGTCGCATCCTGATTGC	*Nco*I
P82	GTCACCATGGACCTGCGTACCTGCTTG	*NcoI*
P83	GTCAGAATTCCACCGGAACTGCTGC	*EcoR*I
P86	TCAGGGTACCTGTTCCGCAAGCTGAC	*KpnI*
P87	ACTGCCATGGCAAGGAGGGAG	*NcoI*
P88	GTCACCATGGTTTAAGCCTGCTGCCTG	*NcoI*
P89	CTGAGAATTCGCTACCGCATCCAACG	*EcoRI*
P93	ACTCGAATTCTTGAGCATGATGTGTG	*Eco*RI
P94	TGACCCATGGCTGTATCCCTTTTATTGTCC	*Nco*I
P95	CTGACCATGGCGGAAGGGCGTTGC	*Nco*I
P96	ACTGGGTACCGTCGTCGAATACGCAC	*Kpn*I
P103	ACTGTCTAGAACACTTCCGCATGTAAC	
P104	ACTGAAGCTTATTAAAGAGACTTGCGCAG	
P120	ACTGGAATTCCAGAAAGGACTGTCGGATG	
P121	ACTGCTGCAGTCAGGCAACATTTCTCAGC	
P122	ACTGTCTAGATAATGATGTCGCTTGCATGG	
P214	AGTCTCTAGATGACGAAAACGATAGC	*XbaI*
P215	AGCTGGTACCGCGATCAGCCTGAGC	*KpnI*
P236	AGTCGGTACCTTGCGGAACTGGAAC	*KpnI*
P237	ACTGTCTAGACAACAAGTTCGTGCAGAG	*XbaI*
P238	AGTCGGTACCTCCAGCGAGAACACG	*KpnI*
P239	ACTGTCTAGAGAACCAGAAGGTCGCGA	*XbaI*
P272	ACTGTCTAGAACTCCAATCCCATCC	*XbaI*
P273	AGTCGGTACCACCGTGCGCAGC	*KpnI*

P280	ACTGTCTAGAATGTTGTGGCTCCC	
P281	ACTGAAGCTTCAGTCGGACGAGCC	
P301	TATAAGCTTAAATATGCCAATAACGCGCG	*HindIII*
P302	TATGGATCCAAGTCGGACGAGCCGGTGCC	*BamHI*
P303	TATGGATCCAATGAATATGCCAATAACGCGCG	*HindIII*
P304	TATGGTACCTCAGTCGGACGAGCCGGTG	*BamHI*

Supplementary Table 2: Plasmids used in this study.

Plasmid	Characteristics	Source/Reference
pBluescript SK(+)	Standard cloning vector	Stratagene
pGPI-SceI	Suicide plasmid vector with I-SceI restriction site, Tp^R	[1]
pDAI-SceI	Plasmid encoding the I-SceI nuclease gene, Tc^R	[1]
pDAIGm-SceI	plasmid encoding the I-SceI nuclease gene, Gm^R	This study
pBBR1MCS-5	Broad-host-range cloning vector, Gm^R	[2]
pYhck	pRK404A carrying the *E. coli yedQ* (*yhck*) gene, Tc^R	[3]
pYedQ	pBBR1MCS-5 carrying the *E. coli yedQ* (*yhck*) gene, Gm^R	This study
pPA5295	Pseudomonas aeruginosa PDE on pBBR1MCS-5, Gm^R	[4]
pBBR-rpfR	pBBR1MCS carrying H111 wild-type rpfR, Cm^R	[5]
pBBR-rpfR^{GGAAF}	pBBR-rpfR harboring D318A and E319A amino acid substitutions, Cm^R	[6]
pBBR-rpfR^{AAL}	pBBR-rpfR harboring an E443A amino acid substitution, Cm^R	[6]
pRpfR^{wt}	pBBR1MCS-5 carrying the H111 wild-type *rpfR* gene, Gm^R	This study
pRpfR^{GGAAF}	pBBR1MCS-5 carrying *rpfR* with mutated GGDEF domain, Gm^R	This study
pRpfR^{AAL}	pBBR1MCS-5 carrying *rpfR* with mutated EAL domain, Gm^R	This study
pBBR2-Bcam1349	pBBR1MCS-2 carrying the *berA* gene, Km^R	[7]
pBerA	pBBR1MCS-5 carrying the *berA* gene, Gm^R	This study
pRpoN	pBBR1MCS-5 carrying the *bca0813* gene, Gm^R	This study
pBerB	pBBR1MCS-5 carrying the *berB* gene, Gm^R	This study
pQE-32	Expression vector for 6xHis-tagged proteins	Qiagen
pQE-RpfR	Expression vector for 6xHis-tagged RpfR	This study
pQE-RpfR^{GGAAF}	Expression vector for 6xHis-tagged RpfR^{GGAAF}	This study
pQE-RpfR^{AAL}	Expression vector for 6xHis-tagged RpfR^{AAL}	This study
pGPI-ΔrpfR	pGPI-SceI based deletion plasmid for *rpfR*, Tp^R	This study
pGPI-ΔrpfF	pGPI-SceI based deletion plasmid for *rpfF*, Tp^R	This study
pGPI-ΔrpfFR	pGPI-SceI based deletion plasmid for *rpfR* and *rpfF*, Tp^R	This study
pGPI-ΔrpoN	pGPI-SceI based deletion plasmid for *rpoN*, Tp^R	This study
pGPI-ΔbceC	pGPI-SceI based deletion plasmid for *bceC*, Tp^R	This study
pGPI-ΔgtaB	pGPI-SceI based deletion plasmid for *gtaB*, Tp^R	This study
pGPI-ΔbepB	pGPI-SceI based deletion plasmid for *bepB*, Tp^R	This study
pGPI-ΔberA	pGPI-SceI based deletion plasmid for *berA*, Tp^R	This study
pGPI-bepB::lacZ	Plasmid for insertion of lacZ downstream of *bepB*, Tp^R	This study
pSUP3535	transcriptional *lacZ* fusion vector, Tc^R	[8]
pGPI2-SceI	pGPI-SceI with modified multiple cloning site, Tp^R	This study
pGPI-rpfR^{GGAAF}	pGPI-SceI based knock-in plasmid for *rpfR*^{GGAAF}, Tp^R	This study
pGPI-rpfR^{AAL}	pGPI-SceI based knock-in plasmid for *rpfR*^{AAL}, Tp^R	This study
pGPI-rpfR^{WT}	pGPI-SceI based knock-in plasmid for *rpfR*^{wt}, Tp^R	This study
pUT18-BerB	Euromedex plasmid with BerB-adenylate cyclase domain 18, Ap100	This study
pUT18C-BerB	Euromedex plasmid with adenylate cyclase domain 18-BerB, Ap100	This study
pKT25-RpfR	Euromedex plasmid with adenylate cyclase domain 25 -BerB, Kn50	This study
pKNT25-RpfR	Euromedex plasmid with RpfR- adenylate cyclase domain 25, Kn50	This study

Gm, gentamycine; Tc, tetracycline, Tp, trimethoprim, Ap, ampicillin, Kn, kanamycin

Supplementary References

1 Flannagan, R. S., Linn, T. & Valvano, M. A. A system for the construction of targeted unmarked gene deletions in the genus *Burkholderia*. *Environ. Microbiol.* **10**, 1652-1660 (2008). https://doi.org:10.1111/j.1462-2920.2008.01576.x

2 Kovach, M. E. *et al.* Four new derivatives of the broad-host range cloning vector pBBR1MCS, carrying different antibiotic-resistance cassettes. *Gene* **166**, 175-176 (1995). https://doi.org:10.1016/0378-1119(95)00584-1

3 Ausmees, N. *et al.* Genetic data indicate that proteins containing the GGDEF domain possess diguanylate cyclase activity. *FEMS Microbiol. Lett.* **204**, 163-167 (2001). https://doi.org:10.1016/s0378-1097(01)00394-9

4 Duerig, A. *et al.* Second messenger-mediated spatiotemporal control of protein degradation regulates bacterial cell cycle progression. *Genes & Dev.* **23**, 93-104 (2009). https://doi.org:10.1101/gad.502409

5 Huber, B. *et al.* Genetic analysis of functions involved in the late stages of biofilm development in *Burkholderia cepacia* H111. *Mol. Microbiol.* **46**, 411-426 (2002). https://doi.org:10.1046/j.1365-2958.2002.03182.x

6 Deng, Y. Y. *et al.* Cis-2-dodecenoic acid receptor RpfR links quorum-sensing signal perception with regulation of virulence through cyclic dimeric guanosine monophosphate turnover. *Proc. Natl. Acad. Sci.* **109**, 15479-15484 (2012). https://doi.org:10.1073/pnas.1205037109

7 Fazli, M. *et al.* The CRP/FNR family protein Bcam1349 is a c-di-GMP effector that regulates biofilm formation in the respiratory pathogen *Burkholderia cenocepacia*. *Mol. Microbiol.* **82**, 327-341 (2011). https://doi.org:10.1111/j.1365-2958.2011.07814.x

8 Mesa, S., Bedmar, E. J., Chanfon, A., Hennecke, H. & Fischer, H. M. *Bradyrhizobium japonicum* NnrR, a denitrification regulator, expands the FixLJ-FixK(2) regulatory cascade. *J. Bacteriol.* **185**, 3978-3982 (2003). https://doi.org:10.1128/jb.185.13.3978-3982.2003
